# The Role of the Extracellular Matrix in Cancer Prevention

**DOI:** 10.3390/cancers17091491

**Published:** 2025-04-29

**Authors:** Stuart G. Baker, Edward R. Sauter

**Affiliations:** 1 Independent Researcher, Columbia, MD 21044, USA; profstu@outlook.com; 2 Division of Cancer Prevention, National Cancer Institute, Bethesda, MD 20892, USA

**Keywords:** extracellular matrix, cancer prevention, hyaluronan, pericytes

## Abstract

The extracellular matrix (ECM) is a major driver of tumorigenesis, yet its role in cancer prevention has received relatively little attention. Here, we discuss studies linking the ECM to cancer initiation with an emphasis on ECM stiffness and remodeling, pericytes, and hyaluronan (hyaluronic acid). We then share our thoughts on how an ECM viewpoint could lead to new insights and directions in cancer-prevention research. Topics discussed include mouse experiments, clinical studies, risk factors, biomarkers for risk prediction or the early detection of cancer, surrogate endpoints, and targets for preventive interventions.

## Simple Summary

The extracellular matrix (ECM) plays a major role in tumorigenesis. Its role in cancer prevention is not well known. Nonetheless, available evidence of how the ECM influences cancer development suggests its use for targeting prevention, risk prediction, early detection, surrogate endpoints, and, understanding risk factors.

## 1. Introduction

**Figure 1:**
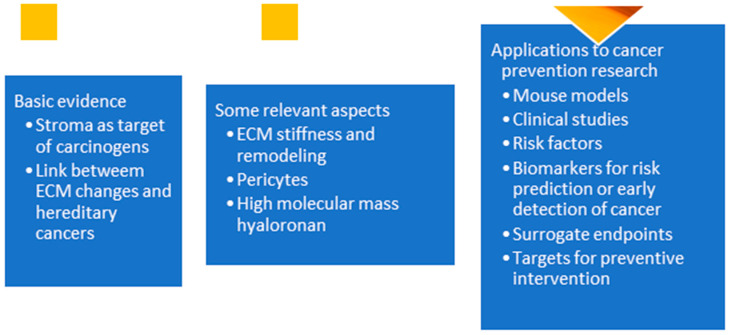
Outline of the paper.

The extracellular matrix (ECM) is a dynamic network of macromolecules that surrounds cells in all solid tissues. It is mainly composed of collagens, elastic fibers, and glycoproteins [1]. Besides providing structural support, the ECM also affects cell proliferation, adhesion, migration, polarity, differentiation, and apoptosis [2]. The ECM is constantly undergoing remodeling involving deposition, degradation, and modification of components [2]. A disruption between ECM production and degradation can lead to excess ECM accumulation, which stiffens the ECM [3]. As summarized in Figure 1, we first discuss basic evidence supporting the role of the ECM in carcinogenesis. Next, we discuss relevant aspects of the ECM for cancer development. Lastly, we discuss six areas for the application of the ECM in cancer-prevention research.

## 2. Basic Evidence

### 2.1. ECM and Tumor Initiation

The stroma, the tissue supporting the epithelial tissue, consists primarily of ECMs. There is strong evidence that changes in the stroma drive the initiation of epithelial tumors. In an important mouse experiment, Maffini et al. [4] showed that a carcinogen can cause epithelial tumors by altering the adjacent stroma. They removed mammary epithelial cells, exposed them to a carcinogen or a control substance, exposed intact stromal fat pads to a carcinogen or a control substance, and reinserted the epithelial cells next to the stromal fat pads. Only the exposure of the carcinogen to the stroma resulted in tumors (Table 1). In all the tables, we computed 95% confidence intervals (CIs) using Wilson’s formula [5], which is appropriate for small sample sizes.

### 2.2. ECM and Hereditary Cancers

Changes in the ECM may also play a major role in the development of hereditary cancers. One example involves the increased risk of cancer among people with a deleterious BRCA1 mutation. Nee et al. [6] found “striking” differences between stromal fibroblasts among people with and without BRCA1 mutations, including elevated levels of matrix metalloproteinase 3 (MMP3), contributing to ECM remodeling [7]. When they transplanted precancerous mammary epithelial cells from BRCA1 carrier mice along with human fibroblasts with or without MMP expression into immunocompromised mice, they found substantially higher tumor incidence among the fibroblasts that expressed MMP3 (Table 2).

Another example involving the role of ECM in hereditary cancer is the high risk of colorectal cancer among patients with inflammatory bowel disease [8], a condition defined by the remodeling of the ECM [9].

## 3. Relevant Aspects of the ECM

### 3.1. ECM Stiffness and Remodeling

Various studies indicate that stiffness and remodeling of the ECM play a major role in tumorigenesis. Mammary epithelial cells in the culture displayed normal epithelial tubulogenesis in soft matrices but showed abnormal tumor-like morphology in stiff matrices [10]. Increasing ECM stiffness in matrix cultures of normal mammary epithelial cells induces malignant phenotypes [11]. Mammary tumor incidence was 3-fold greater in a strain of mice with increased collagen density in comparison with a strain with normal collagen density [12]. Tlsty et al. [13] showed that ECM changes induced by inflammation were necessary and sufficient to create pre-cancerous squamous lung metaplasia.

There is currently active research into the role of ECM in cancer development. One area of interest is mechanotransduction [14], which investigates how the mechanical properties of the ECM affect biochemical signals. Recent articles have discussed how mechanical cues from the ECM may lead to reprogramming of somatic cells based on two pathways [15,16]. One is a physical pathway involving the Linker of Nucleoskeleton and Cytoskeleton (LINC) complex axis. Another is a chemical pathway involving yes-associated protein and the transcriptional coactivator with PDZ-binding motif, abbreviated as YAP/TAZ. Also, the stiffening of the ECM may promote tumor immune evasion [17].

The role of ECM stiffness in carcinogenesis is consistent with the tissue organization field theory (TOFT), which postulates that chronic abnormal interactions between the stroma and the epithelia lead to tumors [18,19]. A key feature of TOFT is the disruption of the underlying morphogenetic field that maintains normal tissue architecture and is analogous to morphogenetic fields that organize tissue morphology in the embryo.

### 3.2. Pericytes

Pericytes may link ECM-induced tissue fibrosis and tumorigenesis. Pericytes are cells that are present at intervals along the walls of capillaries. Originally, they were thought to have a limited role, namely stabilizing blood vessels and angiogenesis. However, their role has expanded into tumor biology [20].

**Figure 2:**
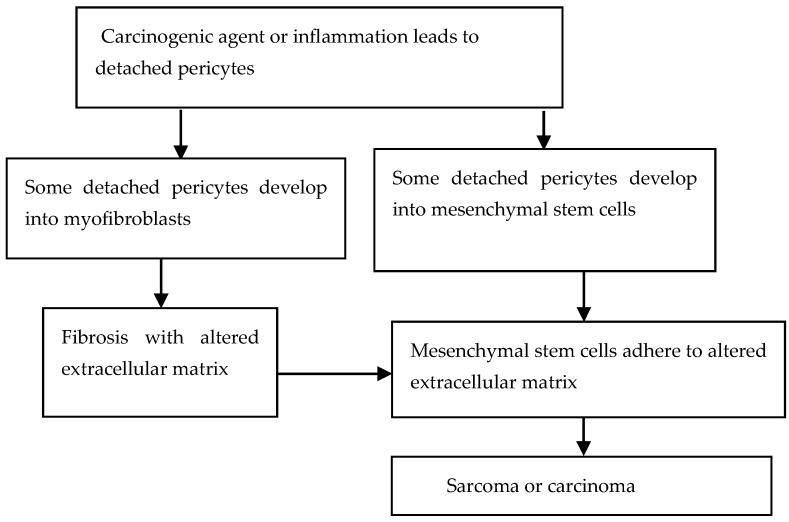
Basic diagram of the detached pericyte hypothesis.

A possible connection between pericytes and cancer is summarized in the detached pericyte hypothesis [21,22]. The detached pericyte hypothesis of tumorigenesis has five related parts, as shown in Figure 2.

(1) Carcinogens or chronic inflammation cause pericytes to detach from the blood vessel walls. Supporting evidence comes from studies of pericytes, inflammation, and tissue injury [23,24].
(2) Some detached pericytes develop into myofibroblasts, which leads to alterations of the ECM. Supporting evidence comes from fate-tracing studies that show that pericytes can develop into myofibroblasts [25].
(3) Some pericytes develop into mesenchymal stems cells (MSCs). Supporting evidence comes from studies that have shown that pericytes can become MSCs [26] and are likely the cells of origin in sarcomas [27].
(4) Some MSCs adhere to the altered ECM. Supporting evidence comes from mouse experiments showing that MSCs adhering to microbeads develop into sarcomas [28].
(5) The altered ECM disrupts normal tissue regulatory controls, causing the MSCs to develop into sarcomas or carcinomas. Supporting evidence comes from studies showing that MSCs can differentiate into epithelial cells in culture [29] and induce epithelial proliferation in the presence of chronic inflammation [30]. Also, transplant studies showed that MSCs can develop into epithelial tumors [31].

Additional evidence supporting the detached pericyte hypothesis comes from fate-tracing studies in zebrafish, showing that neural stem cells, which are precursors of pericytes, play a key role in melanoma initiation [32].

Challenges with research involving pericytes and cancer include difficulties in finding markers that are specific to pericytes, limited knowledge about the heterogeneity of pericytes in different tissues, and a lack of mechanistic studies [33]. Given that metastatic potential may arise early in carcinogenesis [34], the role of pericytes in establishing a pre-metastatic niche [35] may be important for cancer prevention.

### 3.3. Hyaluronan

Hyaluronan (HA), a non-protein component of the ECM, occurs in two forms: low-molecular-mass hyaluronan (LMM-HA) and high-molecular-mass hyaluronan (HMM-HA), with the latter showing anti-inflammatory properties. HMM-HA decreases kidney fibrosis in mice [36], which suggests a possible role for HMM-HA in reducing cancer by reducing fibrosis.

Interestingly, very high molecular mass hyaluronan (vHMM-HA) in naked mole rats likely explains the absence of cancer in these animals. Xenograft experiments showed that genetic alterations preventing the expression of vHHM-HA yielded cells that formed tumors [37]. Also, the increasing expression of vHHM-HA in transgenic mice was found to reduce the incidence of cancer [38].

## 4. New Insights and Directions

The ECM viewpoint can lead to new insights and directions in cancer-prevention research. Topics discussed include mouse experiments, clinical studies, risk factors, biomarkers for risk prediction, surrogate endpoints, and targets for preventive interventions.

### 4.1. Mouse Experiments

The ECM viewpoint can suggest new mouse experiments or mouse experiments deserving replication with a larger sample size. A good example of the latter involves the delayed effect of metformin on cancer incidence. Metformin is a first-line medication for the treatment of diabetes. There is considerable interest in repurposing metformin for cancer prevention because of its safety profile and indications of its effectiveness in preventing cancer [39].

The mechanism by which metformin reduces cancer incidence is not known. One hypothesis is that metformin activates the protein kinase AMPK, which inhibits the mTOR pathway and thereby inhibits cell growth and proliferation [40]. However, the involvement of the mTOR pathway is tenuous. Mouse experiments showed that metformin had no effect on phosphorylation levels of mTOR [40,41]. Metformin requires AMPK activation to reduce fibrosis in mice [42] and reduces markers of fibrosis much less in mice who are genetically deficient in AMPK compared with wild-type mice [43]. Shankaraiah et al. [40] found that mice receiving metformin developed zero tumors per mouse, while control mice developed at least seven tumors per mouse, with the former having lower levels of fibrosis.

Intriguingly, metformin administered after ECM changes may have little effect on cancer incidence. In one experiment, Tajima et al. [41] found that metformin started at baseline reduced cancer incidence in mice with a high-fat diet. In another experiment, metformin started at 30 weeks had no effect on cancer incidence (Table 3).

The delayed start time allowed for the development of nonalcoholic fatty liver disease, a precursor to liver fibrosis [44] that involves remodeling the ECM [45]. These results suggest that metformin may not reduce cancer incidence if administered after ECM remodeling. Because of the importance of these results, we recommend replicating this experiment with a larger sample size.

### 4.2. Clinical Studies

The ECM viewpoint could help select the target population for clinical studies. Individuals at increased risk for cancer are often investigated in cancer prevention studies, given that they have the most to gain from an effective preventive strategy.

Selection of a target population for cancer prevention should involve the same high-risk requirements as used in a trial [46]. Extrapolating from a trial of high-risk individuals to the general population has two major drawbacks [46]. First, it is not clear whether one should extrapolate by assuming invariance in risk difference or invariance in relative risk. Second, the benefit-to-harm ratio may be lower for the general population than the high-risk trial population. Also, a trial for high-risk individuals might be more expensive than a trial in the general population if there is a high cost in identifying high-risk individuals [46].

In terms of biological considerations, high-risk participants with precancerous or noninvasive cancerous lesions constitute a desirable target population; this is a result of the possibility of histologically evaluating the effect of the intervention on the lesions. These lesions include, for example, ductal carcinoma in situ (DCIS), lobular carcinoma in situ (LCIS), atypical hyperplasia of the breast, adenomas of the colon, endometrial intraepithelial neoplasia, actinic keratosis of the skin, and cervical dysplasia.

When designing clinical studies, it is also important to consider the comorbidities that the population may have, and how these may impact treatment response. For example, most clinical studies of metformin, an agent approved to treat type 2 diabetes mellitus, have involved people with diabetes. The results have not been encouraging. A randomized trial of approximately 2000 people with diabetes was inconclusive, with a hazard ratio for cancer incidence of 1.04 and a wide 95% CI of (0.72, 1.52) [47]. Target trial emulation, a method that avoids many biases with other observational studies, found no effect of metformin on cancer incidence among people with diabetes, with an estimated cancer risk difference of −0.2% and a narrow 95% CI of (−1.6, 1.3%) [48]. Results were similar when 83% of the cases were people with diabetes [48].

The ECM viewpoint suggests that metformin may have more positive results in reducing cancer incidence if the target population comprises non-diabetic people. Diabetes causes ECM changes [49], which may negate any effect of metformin on cancer incidence, as suggested by the mouse experiments of Tajmina et al. [41]. Preliminary results with non-diabetic people are encouraging. Higurashi et al. [50] randomized 151 non-diabetic people with previous colorectal adenomas or polyps to low-dose metformin or no treatment. The relative risk for new adenomas identified at colonoscopy 1 year after randomization was 0.60 with 95% CI of (0.39, 0.92). Hosono et al. [51] randomized 26 non-diabetic patients with aberrant crypt foci to metformin or no treatment and found a statistically significant decrease from baseline in the number rectal aberrant crypt foci in the metformin group. This line of evidence can motivate researchers to conduct large clinical studies studying the effect of metformin on cancer incidence among non-diabetic people.

A challenge that arises in conducting cancer-prevention trials is enrolling participants. One possible strategy for increasing enrollment is the following type of encouragement design: randomly selecting participants from an electronic health records database either for follow-up or to offer a prevention approach. Participants who are offered the prevention approach may refuse. Statistical methods for causal inference can adjust for refusers while using randomization to draw rigorous conclusions [52,53]. This design may involve ethical considerations, but it is worth considering. Importantly, any preventive intervention must have a good safety profile, which needs evaluation along with efficacy.

### 4.3. Risk Factors

The ECM viewpoint may help elucidate risk factors for cancer. For example, stiffness and remodeling of the ECM may explain the link between obesity and cancer. Obesity is strongly associated with the increased incidence of at least 13 types of cancers, particularly cancers of the endometrium, liver, colon, pancreas, breast, ovary, and kidney [54,55]. Obesity also increases adipose tissue fibrosis [56]. Mouse adipocytes acquired a fibroblast-like signature in response to a high-fat diet [57], and both mice fed a high-fat diet and mice that were genetically obese had stiffer ECM than control mice [58]. Relating stiffer ECM to carcinogenesis, mammary cell clusters seeded into decellularized ECMs became significantly more disorganized in genetically obese mice in comparison with normal mice [58], and breast tumors from obese patients exhibited more dense connective tissue than breast tumors from lean patients [58].

### 4.4. Biomarkers for Risk Prediction or Early Detection of Cancer

Biomarkers indicating a change in the ECM should be considered for the risk prediction of cancer. The ideal evaluation of biomarkers for risk prediction requires an external validation sample, which is a sample from a population that differs from the population from which the training sample was obtained. Standard statistical metrics are the area under the receiver operating characteristic (ROC) curve, which is denoted as AUC, and calibration plots, which compare predicted and observed frequencies. Decision analytic methods can account for the cost of data collection [59]. Most of the literature on cancer biomarkers focuses on the AUC.

Liver stiffness is a remarkably good predictor of cancer developing many years in the future. A liver cancer risk prediction model based on liver stiffness measured by elastography, age, and platelet count yielded an AUC of 0.78 in an external validation sample at both 2 and 5 years after measurement [60]. A liver stiffness score predicted liver cancer in 5 years with an AUC of 0.85 in a validation sample [61]. The FIB-4 index, which is a biomarker for fibrosis, predicted liver cancer within 10 years with a hazard ratio of 19 with 95% CI of (10 to 37) [62]. Based on a constant odds ratio ROC curve, and approximating the hazard ratio as an odds ratio [63], the AUC would be 0.88.

A potential ECM-related biomarker for risk prediction is hyaluronan expression in the stroma. Corte et al. [64] found higher levels of stromal hyaluronan expression in ductal carcinoma in situ (DCIS) with a microinvasive component than in pure DCIS. However, more rigorous validation is needed with an external validation sample and computation of the AUC.

ECM-related biomarkers may also be useful for the early detection of cancer. For example, investigators have considered ECM proteins in blood specimens for the early detection of lung cancer [65]. Rigorous evaluation should follow a pipeline: (i) diagnostic performance studies with samples from people with diagnosed cancer versus people who have not been diagnosed with cancer; (ii) prediagnostic performance studies with stored specimens; (iii) cancer screening trials [66]. Recently developed methods can substantially reduce the sample size of prediagnostic performance studies [66].

### 4.5. Surrogate Endpoints

The goal of early-phase cancer-prevention trials is to identify safe and effective preventive interventions that be applied in large-scale clinical trials. Because these trials are small with a short duration, they require a surrogate for the true endpoint of cancer mortality. Typical surrogate endpoints are markers for cell proliferation, apoptosis, growth factor expression, oncogene expression, and immune response. However, changes in ECM have received little consideration as surrogate endpoints in these trials. A possible surrogate endpoint based on the ECM is the level of AMPK activation.

Ideally, investigators should validate a surrogate endpoint using multiple trials with both a surrogate and a true endpoint [67]. Two recently developed metrics are the sample size multiplier (the multiple of sample size when using a surrogate endpoint instead of a true endpoint) and true endpoint advantage (the increase in the likelihood of finding statistical significance with a true endpoint instead of a surrogate endpoint) [65].

Validation of AMPK activation as a surrogate endpoint could be feasible with small, precision-focused cancer-prevention trials. One such trial involves metformin in non-diabetic adults with Li–Fraumeni syndrome (LFS) [68]. LFS is characterized by a mutation in the *TP53* gene. The working hypothesis for the trial is that metformin prevents cancer by disrupting mitochondrial DNA. However, because the mutation in the *TP53* gene inhibits AMPK activation [69], metformin has the potential to reduce cancer incidence by increasing AMPK activation. We recommend using the level of AMPK activation as a potential surrogate endpoint along with the true endpoint of 5-year cancer survival.

### 4.6. Targets for Preventive Intervention

The ECM viewpoint can suggest targets for cancer prevention.

#### 4.6.1. Fibrosis Inhibitors

One strategy is to target the various pathways that lead to fibrosis. Zhao et al. [70] listed various antifibrotic drugs with published clinical data or clinical trials that are currently under evaluation in Phase 2 or Phase 3. Two that have received particular attention are nintedanib [71] and pirfenidone [72]. Zhao et al. [70] cautioned that, although most of these drugs demonstrate good antifibrotic properties in clinical trials, side effects often lead to drug discontinuation and reducing adverse effects; these factors pose a great challenge to the use of these drugs.

Another target for reducing fibrosis is the enzyme cluster of differentiation 38 (CD38), which plays a key role in the age-dependent decline of nicotinamide adenine dinucleotide (NAD^+^) [73]. In mouse studies, boosting NAD+ via genetic or pharmacological targeting of CD38 prevented or reduced skin, lung, and peritoneal fibrosis [73,74]. These results suggest investigating CD38 as a target for cancer prevention.

The fibrosis inhibitor losartan, an FDA-approved antihypertensive agent, may also have applications in cancer prevention. Salama et al. [75] randomized 50 patients with liver fibrosis to either losartan or silymarin, an herbal remedy for liver treatment [76]. Among the participants who received a scheduled liver biopsy, regression of fibrosis occurred in 14 of 16 patients randomized to losartan and 2 out of 11 patients randomized to silymarin [77].

#### 4.6.2. Pericyte Targets

There are also potential strategies based on pericytes. For pancreatic ductal cancer, Wu et al. [78] discussed nine possible targets in the stroma, with an emphasis on their effect on pancreatic stellate cells, which are pericytes.

#### 4.6.3. Hyaluronidase Inhibitors

As previously noted, high-molecular-mass hyaluronan (HMM-HA) may have a tumor-protective role [79]. McGuire et al. [79] identified a compound found in some fruits and vegetables, delphinidin, that inhibits hyaluronidases and increases levels of HMM-HA [79]. When they injected delphinidin into tumor-bearing mice, they found that delphinidin slowed the progression of metastasis. There is also evidence that delphinidin suppresses matrix metalloproteinase 9 (MMP-9) [80], which plays a role in fibrosis [81]. Other studies show that delphinidin reduces myofibroblast differentiation and ECM production [82]. Delphinol, an extract from the maqui berry that contains 8.5% delphinidin, was well tolerated in a 3-month trial with no adverse events [83].

## 5. Conclusions

We have provided evidence for the key role of the ECM in cancer initiation and explained how this information could lead to new research directions in cancer-prevention research. The ECM can play an important role in a variety of research opportunities: better understanding of risk factors, better use of surrogate endpoints, better biomarkers for risk prediction or early detection, and better agents for prevention that have good safety profiles. We propose four areas for future research.

First, it is worthwhile considering a clinical trial of metformin in non-diabetic people. Metformin has an excellent safety profile. Based on mouse studies and preliminary trials in non-diabetic people, we think that metformin has promise as a cancer-prevention agent in non-diabetic people.

Second, investigators performing precision cancer-prevention trials should collect data on both possible surrogate endpoints and a true endpoint. A possible surrogate endpoint related to the ECM is a measure of AMPK activation. Data on both surrogate and true endpoints in multiple trials provide useful information for evaluating the surrogate endpoint. A good surrogate endpoint could greatly shorten future cancer prevention studies.

Third, investigators should consider risk-prediction markers and markers for the early detection of cancer that reflect changes in the ECM. ECM markers in the blood are good candidates for cancer screening because they are noninvasive.

Fourth, investigators should more thoroughly investigate the role of the ECM in DCIS. Approximately, 20% to 60% of women with DCIS will progress to invasive disease [76]. There is no clear way of determining which women will develop invasive disease, and many women opt for bilateral mastectomy with or without reconstruction, with the attendant risks of complications and costs. The goal is a safe and effective strategy based on the ECM that provides a nonsurgical approach to mitigate the risk of invasive cancer. Research can involve comparisons of ECM stiffness, pericytes, and hyaluronan levels in women with and without DCIS.

## Figures and Tables

**Table 1:** Results of mouse experiment by Maffini et al. [4] with 95% CIs computed using Wilson’s formula [5].

Carcinogen Applied to Stroma	Carcinogen Applied to Re-Inserted Epithelial Tissue	Number of Mice	Fraction with Tumors (95% CI)
No	No	6	0.00 (0.00, 0.39)
	Yes	10	0.00 (0.00, 0.29)
Yes	No	13	0.77 (0.50, 0.92)
	Yes	8	0.75 (0.41, 0.93)

**Table 2:** Results from mouse experiment by Nee et al. [6] with 95% CIs computed using Wilson’s formula [5].

Experimental Groups	Number of Mice	Fraction with Tumors (95% CI)
BRCA1 Mammary cells	12	0.33 (0.14, 0.61)
BRCA1 Mammary cells + fibroblasts	12	0.67 (0.39, 0.86)
BRCA1 Mammary cells + fibroblasts + MMP3	12	1.00 (0.76, 1.00)

**Table 3:** Results of mouse experiment by Tajima et al. [41]. The 95% CIs were computed using Wilson’s formula [5].

Experiment	Experimental Groups	Number of Mice	Fraction with Tumors (95% CI)
1	Control	16	0.69 (0.44, 0.86)
	Metformin at start	17	0.29 (0.13, 0.53)
2	Control	4	0.75 (0.30, 0.95)
	Metformin delay	6	0.83 (0.44, 0.97)

## Data Availability

No new data were generated in the preparation of this manuscript.
